# Influence of Angiotensin II Subtype 2 Receptor (AT_2_R) Antagonist, PD123319, on Cardiovascular Remodelling of Aged Spontaneously Hypertensive Rats during Chronic Angiotensin II Subtype 1 Receptor (AT_1_R) Blockade

**DOI:** 10.1155/2012/543062

**Published:** 2012-03-04

**Authors:** Emma S. Jones, M. Jane Black, Robert E. Widdop

**Affiliations:** ^1^Department of Pharmacology, Monash University, Clayton, VIC 3800, Australia; ^2^Department of Anatomy and Cell Biology, Monash University, Clayton, VIC 3800, Australia

## Abstract

Cardiac AT_2_R expression is upregulated in the normal process of aging. In this study we determined the contribution of AT_2_R to chronic antihypertensive and remodelling effects of AT_1_R blockade in aged hypertensive rats. Adult (20 weeks) and senescent (20 months) spontaneously hypertensive rats (SHRs) were treated with either the AT_1_R antagonist, candesartan cilexetil (2 mg/kg/day), the AT_2_R antagonist, PD123319 (10 mg/kg/day), or a combination of the 2 compounds. Mean arterial pressure (MAP) and left ventricular volume were markedly decreased by candesartan cilexetil, however, simultaneous treatment with PD123319 had no additional effect on either parameter. Perivascular fibrosis was significantly reduced by candesartan cilexetil in aged animals only, and this effect was reversed by concomitant PD123319 administration. Vascular hypertrophy was reduced by candesartan cilexetil, and these effects were reversed by simultaneous PD123319. These results suggest that AT_2_R stimulation does not significantly influence the antihypertensive effect of chronic AT_1_R blockade, but plays a role in the regulation of vascular structure. The severe degree of cardiac perivascular fibrosis in senescent animals was regressed by AT_1_R blockade and this effect was reversed by simultaneous AT_2_R inhibition, demonstrating an antifibrotic role of AT_2_R stimulation in the aging hypertensive heart.

## 1. Introduction

The incidence of hypertension, cardiac hypertrophy, and heart failure increases significantly with aging [1], and age-related structural adaptations may contribute to deteriorating function of the cardiovascular system. The aging heart is characterised by myocyte loss, hypertrophy of remaining cells, and exaggerated accumulation of extracellular (ECM) proteins [1, 2], which is associated with increased incidence of both contractile and conductile dysfunction of senescent hearts [2]. In addition, structural modifications of the aorta and coronary vasculature, particularly involving hypertrophy/hyperplasia of smooth muscle cells and increased collagen deposition within and surrounding the media of vessels [3], result in arterial stiffening, alterations in vascular permeability, and deterioration of coronary haemodynamics [4].

Ang II is known to promote cardiovascular hypertrophy and fibrosis via AT_1_R stimulation [5, 6], whereas the role of AT_2_R has been less conclusively defined [7]. AT_2_R activation is thought to oppose AT_1_R-mediated hypertrophic and fibrotic effects; however, studies in transgenic mouse models of targeted deletion [8, 9] or overexpression [10] of AT_2_R have reported contrasting effects on cardiovascular structure, emphasising the need for further pharmacological investigation and elucidation of AT_2_R function.

AT_1_R antagonists increase circulating levels of Ang II, which may stimulate unopposed AT_2_R and potentially contribute to the effects of AT_1_R blockade [11]. We have previously shown that impaired *in vitro* AT_2_R-mediated relaxation in SHRs was restored by antihypertensive treatment [12]. Furthermore, AT_2_R stimulation may influence cardiovascular function and structure during chronic AT_1_R blockade [13–15]. These studies have been performed in animal models of genetic hypertension or following cardiovascular infarct and have deduced various degrees of AT_2_R-mediated antihypertrophic and antifibrotic effects, depending on the study.

Importantly, although cardiac AT_2_R expression is relatively low in the adult rat heart [16], expression may be upregulated in certain disease states and has been particularly associated with conditions of increased fibrosis [17], cardiac hypertrophy [18], heart failure [19], and also with increasing age [20, 21]. Moreover, increased myocardial angiotensinogen and ACE indicate that intracardiac production of Ang II may also be potentiated with senescence [22].

Given the possibility of augmented cardiac RAS activity with increased age, and also the fact that chronic AT_1_R blockade increased longevity in rodent models of aging and was associated with cardiovascular protective effects [23, 24], we reasoned that a greater AT_2_R contribution to AT_1_R inhibition may be manifest in the aged hypertensive state. Therefore, the aims of this study were to determine the contribution of the AT_2_R to the antihypertensive and cardiovascular remodelling effects of chronic AT_1_R blockade in aged SHRs.

## 2. Materials and Methods

### 2.1. Animals and Treatment

Male SHRs (12 weeks) were obtained from the Animal Resource Centre, Western Australia and were maintained on a 12-hour day/night cycle with free access to food and water until animals were either 20 weeks or 20 months of age. Senescent animals were used at 20 months, as at this age, SHRs display many of the features of hypertensive and age-related cardiac remodelling (including cardiovascular hypertrophy and fibrosis) but are yet to complete the transition to heart failure [25].

Radiotelemetry transmitters (TA11PA-C40, Data Sciences) were inserted into the abdominal aorta of SHRs under isoflurane anaesthesia (2–4%, O_2_), as previously described [26]. Animals were allowed to recover for 1 week, after which time a continuous baseline recording of MAP and HR was made for a further week. Animals were then given the AT_1_R antagonist, candesartan cilexetil (2 mg/kg/day), its vehicle, or the nonangiotensin antihypertensive, hydralazine (30 mg/day), in drinking water. At the same time, senescent SHRs were also briefly anaesthetised with isoflurane, and osmotic mini pumps containing either PD123319 (10 mg/kg/day) or saline vehicle were inserted into a subcutaneous pocket formed between the scapulae. Doses of candesartan cilexetil and PD123319 were based on previous studies performed in senescent Wistar Kyoto rats [26]. Adult SHRs were treated for 2 weeks with candesartan cilexetil (2 mg/kg/day), before implantation of osmotic mini pumps, such that animals received the combination of 6-week candesartan cilexetil and 4-week PD123319 treatment. In senescent SHRs, all drug treatments were initiated simultaneously and continued for 4 weeks duration. MAP and HR were recorded continuously during the entire 4- or 6-week treatment period. Treatment groups were as follows:

*Adult (20 weeks) SHRs*

- control (*n* = 6),
- candesartan cilexetil alone (*n* = 7),
- candesartan cilexetil + PD123319 (*n* = 7),
- PD123319 alone (*n* = 7).

*Senescent (20 months) SHRs*

- control (*n* = 10),
- candesartan cilexetil alone (*n* = 9),
- candesartan cilexetil + PD123319 (*n* = 9),
- PD123319 alone (*n* = 4),
- hydralazine (*n* = 7).

### 2.2. Determination of Plasma Ang II Levels

At the end of the treatment period, a sample of blood was collected directly from the catheterised aorta of each animal into chilled, heparinised tubes, and then centrifuged at 4000 rpm at 4°C for 10 minutes to isolate plasma. The resultant plasma sample was stored at −80°C for later analysis. Ang II concentrations were analysed in duplicate by RIA as described previously [27]. Briefly, plasma (100 *μ*L) was equilibrated with antibody raised in rabbit against Ang II, which was N-terminally conjugated to bovine thyroglobulin. Monoiodinated ^125^I- Ang II tracer (10 000 cpm in 100 *μ*L) was added and allowed to equilibrate for 16 hrs at 4°C, whereupon bound and free phase was separated using Dextran 10-coated charcoal and centrifugation. Sensitivity was 3.5 pg/mL. Intra- and interassay variabilities were 6.4 and 12.0%. Cross reactivity to other angiotensins were Ang I = 0.52%, Ang (1–7) = 0.01%, and to all other pertinent hormones less than 0.10%.

### 2.3. Perfusion Fixation

After 4 or 6 weeks treatment, animals were anaesthetised (ketamine/xylazine; 100 mg/10 mg per kg), and the abdominal aorta briefly ligated to enable removal of the radiotelemetry probe. A catheter was inserted into the abdominal aorta, and a sample of blood was collected into a heparinised tube. Heparin sodium (1 IU/g body weight), papaverine hydrochloride (1.2 mg/rat), and potassium chloride (60 mM in 0.1 mL) were administered via the catheterized aorta to prevent blood from clotting, maximally dilate blood vessels, and arrest the heart in diastole, respectively. Organs were cleared of blood with physiological saline, and then perfusion fixed with 4% paraformaldehyde in 0.1 M phosphate buffer. Perfusion pressure was maintained at a pressure corresponding to the *in vivo* systolic pressure of adult and aged SHRs by use of a perfusion apparatus attached to a sphygmomanometer. Hearts and blood vessels were then excised and stored immersed in paraformaldehyde at 4°C for later processing.

### 2.4. Cardiac Remodelling

Both left and right atria were removed from fixed hearts, and the remaining left ventricle (LV), right ventricle (RV), and septum were weighed. Hearts were then cut into approximately twelve 1.5 mm thick slices using a razor blade slicing device. Each slice was then placed on a light table, images were captured using a video camera module (Sony, XC-77CE CCD, Japan) displayed on a monitor, and analysed using imaging computer software (Microscope Computed Imaging Device M4 (MCID), Imaging Research, Canada). Sampled cross-sectional areas of the LV, RV, and both LV and RV chambers were then multiplied by slice thickness to calculate the volume of each sampled area. Total volumes of LV, RV, LV chamber and RV chamber of each heart were determined by adding measurements taken from heart slices throughout the entire heart. Ventricular weight and volume measurements were normalized to body weight for each animal.

### 2.5. Interstitial and Perivascular Fibrosis in the Heart

After heart volumes had been determined, five 1.5 mm heart slices from each animal were embedded in paraffin, sectioned at 5 *μ*m and stained for collagen with 0.001% Picrosirius Red. Each section was viewed under a light microscope (Olympus, BH-2, Japan) with a video camera module interfaced to a computer. Images were displayed onto a monitor and analysed using imaging computer software (MCID). All sections were examined under ×200 magnification.

The area of interstitial fibrosis in 6 fields of view of the LV per section and 2 fields of view of the RV per section were sampled, and the percentage of fibrosis within each sampled area was averaged for each animal. Collagen volume fraction (%) was calculated by determining the area stained for collagen as a percentage of the total area of sampled tissue, per field of view. Perivascular fibrosis was investigated in the LV only. Two intramyocardial arterioles (measuring 100–200 *μ*m in diameter) were randomly selected per section. The cross-sectional area (CSA) of adventitia (representing perivascular fibrosis), media, and lumen were determined and averaged for each animal. Perivascular fibrosis was normalised to lumen area. As an index of microvascular remodelling, media-to-lumen ratio of intramyocardial vessels was determined from CSA measurements and averaged for each animal.

### 2.6. Aortic Hypertrophy

Segments of fixed vessel were dissected from the thoracic portion of the aorta of each animal, embedded in epon-araldite, cut at 1 *μ*m, and stained with toluidine blue. Each section was viewed under a light microscope and analysed using imaging computer software (MCID). All sections were examined under ×100 magnification. CSA of the media was determined for each vessel and normalised to lumen CSA.

### 2.7. Statistics

The effect of drug treatments on MAP and HR over time was assessed by one- or two-way analysis of variance (ANOVA) with repeated measures, as appropriate. Differences in morphometric data between treatments were determined using one-way ANOVA, followed by a Bonferroni post hoc test. Results are expressed as mean ± standard error of the mean (SEM). Statistical significance was accepted as a probability of **P** < 0.05.

## 3. Results

### 3.1. Body Weight and Plasma Ang II

Body weight was not affected by any drug treatments (Table 1). Compared to age-matched untreated animals, plasma Ang II levels were increased more than 7- and 3-fold by candesartan cilexetil treatment, either alone or in combination with PD123319, in adult and senescent SHRs, respectively (Table 1). Both PD1231319 alone and hydralazine had no effect on plasma Ang II levels (Table 1).

### 3.2. Blood Pressure and Heart Rate in Senescent SHRs

**Figure 1:**
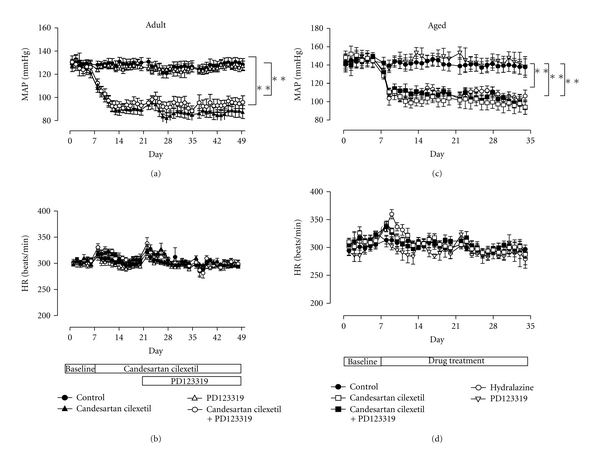
Radiotelemetry recordings of (a) MAP and (b) HR of adult SHRs, at baseline and during treatment with vehicle (control, *n* = 6), candesartan cilexetil (2 mg/kg/day) alone (*n* = 7) or in combination with PD123319 (10 mg/kg/day, *n* = 7), or PD123319 alone. Analogous radiotelemetry recordings of (c) MAP and (d) HR of senescent SHRs, at baseline and during treatment with vehicle (control, *n* = 10), candesartan cilexetil alone (*n* = 9) or in combination with PD123319 (*n* = 9), PD123319 alone (*n* = 4), or hydralazine (30 mg/kg/day, *n* = 7). **P < 0.01 versus control (2-way ANOVA).

Candesartan cilexetil caused a marked reduction in MAP compared to control animals, and administration of PD123319 did not reverse this antihypertensive effect, in either adult or senescent animals (Figures 1(a) and 1(c)). MAP was unaffected by PD123319 alone. In senescent SHRs, hydralazine caused a reduction in MAP that was similar in magnitude to that caused by AT_1_R blockade (Figure 1(c)). Candesartan cilexetil and hydralazine increased HR at the initiation of antihypertensive treatment in both age groups, which most likely represents a reflex tachycardia which persisted for 2-3 days until resetting of the baroreflex occurred. HR after this initial period was unaffected by treatments (Figures 1(b) and 1(d)).

### 3.3. Cardiac Remodelling

**Figure 2:**
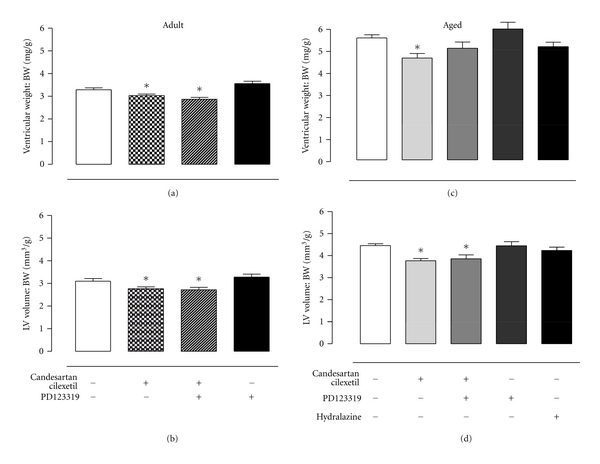
(a) Ventricular weight to body weight (BW) ratio and (b) left ventricular (LV) volume to body weight ratio of adult SHRs, at baseline and during treatment with vehicle (control, *n* = 4), candesartan cilexetil (2 mg/kg/day) alone (*n* = 4), or in combination with PD123319 (10 mg/kg/day, *n* = 4), and PD123319 alone (*n* = 7). (c) Ventricular weight to BW ratio and (d) LV volume to BW ratio of senescent SHRs, at baseline and during treatment with vehicle (control, *n* = 10), candesartan cilexetil (2 mg/kg/day) alone (*n* = 9), or in combination with PD123319 (10 mg/kg/day, *n* = 9), PD123319 alone (*n* = 4), or hydralazine (30 mg/kg/day, *n* = 7). *P < 0.05 versus control (1-way ANOVA).

Ventricular weight (Table 1), ventricular weight to body weight ratio (Figure 2(a)) and LV volume to body weight ratio (Figure 2(b)) of adult SHRs were reduced by candesartan cilexetil; however, this antihypertrophic action was not further influenced by simultaneous AT_2_R inhibition. Similarly, ventricular weight (Table 1), ventricular weight to body weight ratio (Figure 2(c)), and LV volume to body weight ratio (Figure 2(d)) of senescent SHRs were also decreased by AT_1_R blockade. Furthermore, the regression of both ventricular weight and ventricular weight to body weight ratio were partially reversed by concurrent PD123319 treatment, such that these indices were not significantly different from control values. There were no effects of drug treatments on RV, LV chamber or RV chamber, volume to body weight ratios (data not shown).

### 3.4. Interstitial and Perivascular Fibrosis

**Figure 3:**
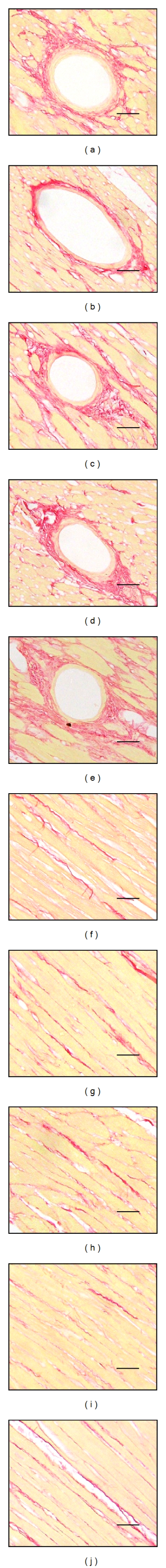
Representative light micrographs of cardiac (a–e) perivascular and (f–j) interstitial fibrosis in senescent SHRs treated with (a, f) vehicle (control), (b, g) candesartan cilexetil (2 mg/kg/day), (c, h) candesartan cilexetil in combination with PD123319 (10 mg/kg/day), (d, i) PD123319 alone, or (e, j) hydralazine (30 mg/kg/day). Scale bar = 50 *μ*m.

**Figure 4:**
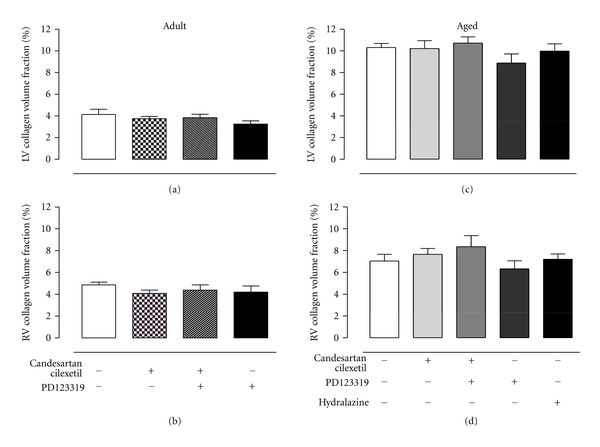
Mean data of interstitial collagen volume fraction of (a) left and (b) right ventricles of adult SHRs treated with vehicle (control, *n* = 6), candesartan cilexetil (2 mg/kg/day) alone (*n* = 7) or in combination with PD123319 (10 mg/kg/day, *n* = 7) or PD123319 alone. Interstitial collagen volume fraction of (c) left and (d) right ventricles of senescent SHRs treated with vehicle (control, *n* = 10), candesartan cilexetil alone (*n* = 9) or in combination with PD123319 (*n* = 9), PD123319 alone (*n* = 4), or hydralazine (30 mg/kg/day, *n* = 7).

**Figure 5:**
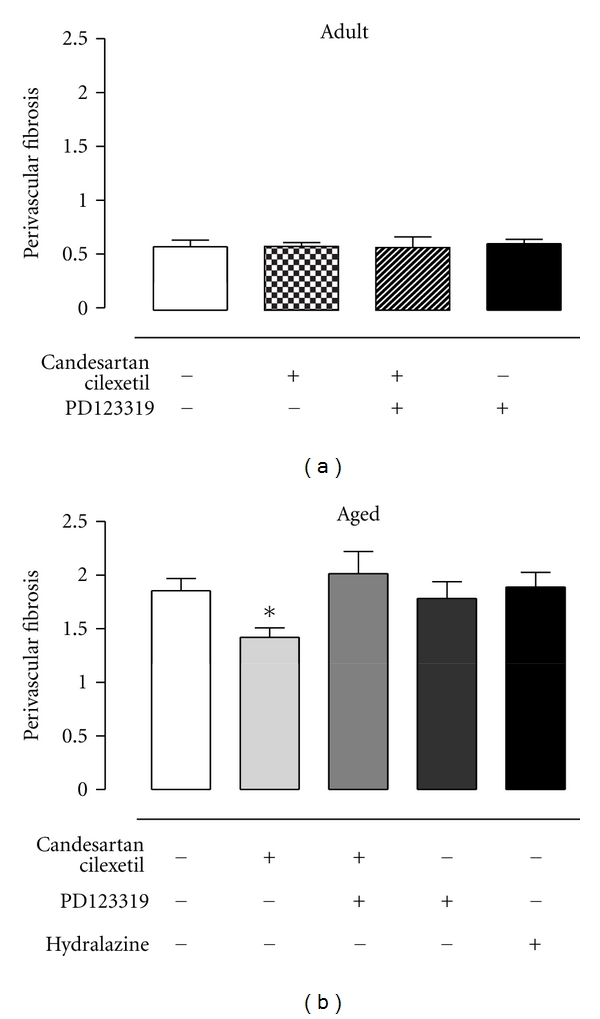
(a) Perivascular fibrosis of adult SHRs treated with vehicle (control, *n* = 6), candesartan cilexetil (2 mg/kg/day) alone (*n* = 7) or in combination with PD123319 (10 mg/kg/day, *n* = 7), or PD123319 alone (*n* = 7). (b) Perivascular fibrosis of senescent SHRs treated with vehicle (control, *n* = 10), candesartan cilexetil alone (*n* = 9) or in combination with PD123319 (*n* = 9), PD123319 alone (*n* = 4), or hydralazine (30 mg/kg/day, *n* = 7). Perivascular fibrosis of intramyocardial arterioles, calculated as cross-sectional area of adventitia to lumen ratio. *P < 0.05 versus control (1-way ANOVA).

Representative light micrographs of perivascular and interstitial fibrosis of senescent SHRs are shown in Figure 3. Group data shows that neither left (Figures 4(a) and 4(c)) nor right (Figures 4(b) and 4(d)) ventricular interstitial fibrosis of adult and senescent SHRs were altered by any drug treatments. Likewise, perivascular fibrosis of adult SHRs was not influenced by AT_1_ or AT_2_R inhibition (Figure 5(a)). In contrast, perivascular fibrosis was significantly decreased by ~28% in senescent SHRs receiving candesartan cilexetil, and this effect was completely reversed by simultaneous AT_2_R blockade (Figure 5(b)).

### 3.5. Vascular Hypertrophy

**Figure 6:**
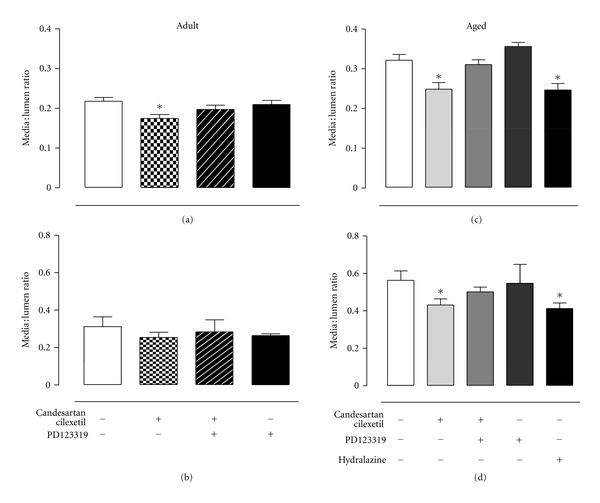
Media to lumen ratio of (a) aortic and (b) intramyocardial vessels of adult SHRs treated with vehicle (control, *n* = 6), candesartan cilexetil (2 mg/kg/day) alone (*n* = 7) or in combination with PD123319 (10 mg/kg/day, *n* = 7) or PD123319 alone (*n* = 7). Media to lumen ratio of (c) aortic and (d) intramyocardial vessels of senescent SHRs treated with vehicle (control, *n* = 10), candesartan cilexetil alone (*n* = 9) or in combination with PD123319 (*n* = 9), PD123319 alone (*n* = 4), or hydralazine (30 mg/kg/day, *n* = 7). *P < 0.05 versus control (1-way ANOVA).

Media to lumen ratios of aortic vessels in both adult and senescent SHRs (Figures 6(a) and 6(c)) and intramyocardial vessels of senescent SHRs (Figure 6(d)) were decreased by candesartan cilexetil, and this antihypertrophic effect of AT_1_R blockade was reversed by concomitant PD123319 administration. Hydralazine also caused a significant reduction in media-to-lumen ratios of aortic (Figure 6(b)) and intramyocardial (Figure 6(d)) vessels of senescent SHRs.

## 4. Discussion

We have shown for the first time, a role for AT_2_R in cardiac and vascular remodelling in a clinically relevant animal model of aging and hypertension. Notably, AT_2_R stimulation by endogenously raised Ang II levels contributed to the cardiac antifibrotic and vascular antihypertrophic effects of chronic AT_1_R blockade. Thus, this study highlights the importance of AT_2_R in the chronic regulation of cardiovascular structure in the aging hypertensive heart and vasculature.

Candesartan cilexetil caused a marked reduction in MAP in both adult and senescent SHRs, which was not further affected by AT_2_R blockade. These results imply that stimulation of the AT_2_R does not significantly influence chronic blood pressure regulation and is consistent with other long-term studies that showed either no [13, 14, 28] or minimal [15] reversal of AT_1_R-blocker- (ARB-) mediated blood pressure-lowering by simultaneous AT_2_R blockade in SHRs. These findings are in direct contrast to the acute setting, in which the antihypertensive effect of ARB compounds was reversed by simultaneous AT_2_R blockade with PD123319 [29–31]. In addition, acute stimulation of AT_2_R has also been shown to lower blood pressure in rats, supporting a role for AT_2_R in acute blood pressure regulation [32–35].

Since both human and animal studies have shown circulating Ang II and renin levels to be reduced with increasing age [22, 36, 37], it is possible that the absence of AT_2_R-mediated actions on blood pressure in aged SHRs is due to depressed systemic RAS activity in senescence. However, in this study we have shown a similar inability of PD123319 to reverse the ARB-induced reduction in blood pressure in both adult and senescent rats. Moreover, even though baseline levels of Ang II are relatively low in aged SHRs compared to adult SHRs (~3-fold lower than adult SHRs, Table 1), AT_1_R inhibition caused an increase in plasma Ang II of 3-4-fold, suggesting that the RAS is still sensitive to perturbation in aged SHRs. Moreover, local tissue production of Ang II has been shown to be elevated in aged humans [6] and rodents [38]. Thus it is more likely that the inability of PD123319 administration to reverse ARB-induced antihypertensive effects in the current context reflects a subtle influence of AT_2_R stimulation on blood pressure regulation being masked by the dominant impact of AT_1_R blockade.

Candesartan cilexetil decreased indices of cardiac growth of adult rats (ventricular weight, and LV volume to body weight ratios), and PD123319 administration had no further influence on these parameters, suggesting no major role for AT_2_R in cardiac hypertrophy. Other studies in hypertensive models have also reported that PD123319 administration did not significantly reverse cardiac hypertrophy [15, 19, 28], and additionally, AT_2_R were deduced to have no major function in the regulation of cardiac mass from studies in transgenic mice models of targeted deletion or cardiac-specific over expression of AT_2_R [10, 39, 40]. In contrast, a dependence on AT_2_R for ARB-mediated cardiac remodelling following MI has been demonstrated in rats [13] and AT_2_R knock out mice [40]. These mismatches in reported AT_2_R influence on cardiac hypertrophy most likely reflect the gross measures of cardiac hypertrophy made in the majority of studies, as heart mass is commonly employed as a surrogate marker for cardiac hypertrophy (i.e., increased cardiomyocyte size) but is unable to distinguish between changes in proportion of specific components within the heart.

Indeed, ventricular weight and LV volume to body weight ratios were also reduced by AT_1_R blockade in senescent SHRs; however, in these aged animals, simultaneous AT_2_R inhibition caused a partial reversal of ventricular weight to body weight ratio. Given that LV volume is heavily influenced by changes in cardiomyocyte area [2], and that LV volume was not influenced by AT_2_R blockade, PD123319-mediated reversal of heart weight to body weight ratio most likely reflects changes in the nonmyocyte components of the heart, rather than a true effect on cardiac hypertrophy. Indeed, we have shown that perivascular fibrosis of coronary microvessels is decreased by AT_1_R blockade and that this effect is reversed by concomitant AT_2_R blockade, but only in senescent hearts. We have previously shown a similar mismatch between LV volume and ventricular weight following AT_1_R blockade in senescent normotensive WKY rats [26], which also coincided with a cardiac AT_2_R-mediated antifibrotic action. Thus the PD122319-mediated increase in ventricular weight to body weight ratio during AT_1_R blockade may in fact be due to inhibition of an AT_2_R-mediated antifibrotic action in senescent SHRs.

Surprisingly, candesartan cilexetil did not reduce interstitial fibrosis in either adult or senescent SHRs. In the present study, particularly high levels of LV and RV interstitial fibrosis (collagen volume fraction ~7–10%) were seen in control senescent animals. These relatively high levels of interstitial fibrosis in aged hearts are entirely consistent with previous studies [15, 20], and contrast with the degree of fibrosis in adult hypertensive SHRs (interstitial collagen volume fraction ~4-5%). We have previously shown that an identical treatment regime markedly reduced interstitial fibrosis from similar levels in aged normotensive WKY (interstitial collagen volume fraction ~4-5%), and this effect was also reversed by PD123319 [26]. Thus in the current study, it appears that the inability of candesartan cilexetil to reduce interstitial fibrosis in both adult and senescent SHRs, results from modifications of the ECM specifically related to hypertension, rather than particularly high pretreatment basal levels of fibrosis. Indeed, collagen cross-linking has been shown to be augmented by hypertension [41, 42], and increased cross-linking is associated with diminished susceptibility of the ECM to proteolytic degradation [43]. Alteration in ECM degradation due to increased glycation cross-linking has been associated with decreased activity of proteolytic enzymes such as matrix metalloproteinase 1 and 2 (MMP-1 and MMP-2) [44], and findings of decreased activity of MMP-1 and MMP-2 by 40–45% in aged, hypertensive rats [45] further support the notion of impaired collagen degradative mechanisms in senescent hypertensive hearts.

On the other hand, the antifibrotic action of AT_2_R stimulation on perivascular fibrosis in senescent rats, as demonstrated in the current investigation, is in accordance with other chronic *in vivo* studies, which have also shown increased cardiac fibrosis during AT_2_R blockade [15, 19, 26]. Similarly, investigators who have used either targeted deletion [8, 40, 46] or cardiac overexpression [10] of AT_2_R in mice have also deduced an antifibrotic role of the AT_2_R. Importantly, cardiac fibrosis induced by circulating humoral factors such as Ang II, typically initiates around blood vessels and then progresses to infiltrate interstitial areas, resulting in a temporal divergence in onset (and thus conceivably also of regression) of the two types of fibrosis related to location [47]. In this context, it is possible that interstitial fibrosis may have been reduced by a longer duration treatment with an AT_1_R antagonist, as has been reported by other investigators following AT_1_R blockade for 12 weeks [15].

In the current study, media-to-lumen ratio of both aortae and coronary vessels was decreased by candesartan cilexetil treatment and also by hydralazine in aged SHRs. This vascular antihypertrophic effect is consistent with previous reports that increased medial thickness due to hypertrophy/hyperplasia of smooth muscle cells is closely related to pressure [48, 49]. However, the other major modification of vascular structure that occurs in hypertension and senescence is an increase in vascular collagen content, the levels of which have been shown to be poorly associated with MAP, but sensitive to AT_1_R inhibition [48]. As the vascular antihypertrophic effect of candesartan cilexetil was reversed, but MAP was unchanged by PD123319, it is reasonable to suggest that the effect of AT_2_R inhibition on vascular remodelling was pressure-independent and thus may be via a reduction in vascular collagen. Furthermore, such a pressure-independent influence of AT_2_R on collagen accumulation in aged SHRs is consistent with effects on perivascular fibrosis in this study, which were decreased by AT_1_R blockade but unaffected by hydralazine, despite both treatments resulting in similar reductions in MAP.

A limitation of this study was that we did not confirm that the reversal of ARB-mediated antifibrotic effects by PD123319 is solely via AT_2_R mechanisms. Indeed, we [50] and others have shown that in certain situations, PD123319 may inhibit the effects of Ang 1–7, which is considered the endogenous ligand for the *Mas* receptor (*Mas*R). However, we have also recently reported that Ang 1–7 shows significant AT_2_R binding [51], which is consistent with PD123319-mediated reversal of Ang 1–7 effects being due to inhibition of AT_2_R rather than a nonselective action at MasR. Nevertheless, definitive elucidation of this issue regarding selectivity of PD123319 requires future determination of *Mas*R binding.

The present study demonstrates an important role for AT_2_R in cardiovascular remodelling in senescent SHRs, as evidenced by the fact that AT_2_R inhibition with PD123319 reversed ARB-mediated regression of perivascular fibrosis in aged SHRs only. Furthermore, we have shown an inhibitory influence of AT_2_R in vascular remodelling, which was apparent in both adult and senescent SHRs, and occurred despite a lack of AT_2_R-mediated effects on blood pressure. Given that our population is aging and that AT_1_R antagonists are commonly used antihypertensives in this demographic, this study provides information regarding the functional relevance of AT_2_R in the physiologically relevant setting of hypertension and senescence, which may have important implications for optimising cardiovascular therapeutics in the elderly.

## Figures and Tables

**Table 1:** Effect of drug treatments on body weight, ventricular weight, and plasma Ang II of adult and senescent SHRs.

	Control	Candesartan cilexetil	Candesartan cilexetil + PD123319	PD123319	Hydralazine
Body weight (g)					
Adult SHRs	411 ± 11	415 ± 5	410 ± 5	414 ± 3	—
Senescent SHRs	417 ± 9	427 ± 7	428 ± 10	399 ± 9	419 ± 11
Ventricular weight (mg)					
Adult SHRs	1423 ± 23	1229 ± 39*	1207 ± 30*	1474 ± 48	—
Senescent SHRs	2114 ± 74	1796 ± 85*	1962 ± 86	2136 ± 163	1952 ± 49
Plasma Ang II (pg/mL)					
Adult SHRs	220 ± 100	1597 ± 234*	2785 ± 817*	68 ± 18	—
Senescent SHRs	80 ± 10	383 ± 89*	271 ± 51*	64 ± 23	91 ± 16

Values are mean ± SEM. *P < 0.05 versus age-matched control (1-way ANOVA).
